# No sex difference in an everyday multitasking paradigm

**DOI:** 10.1007/s00426-018-1045-0

**Published:** 2018-07-02

**Authors:** Marco Hirnstein, Frank Larøi, Julien Laloyaux

**Affiliations:** 1 Department of Biological and Medical Psychology, University of Bergen, Jonas Lies vei 91, 5009 Bergen, Norway; 2 NORMENT-Norwegian Center of Excellence for Mental Disorders Research, University of Oslo, Oslo, Norway; 3 Psychology and Neuroscience of Cognition Research Unit, University of Liège, Liège, Belgium

## Abstract

According to popular beliefs and anecdotes, females best males when handling multiple tasks at the same time. However, there is relatively little empirical evidence as to whether there truly is a sex difference in multitasking and the few available studies yield inconsistent findings. We present data from a paradigm that was specifically designed to test multitasking abilities in an everyday scenario, the computerized meeting preparation task (CMPT), which requires participants to prepare a room for a meeting and handling various tasks and distractors in the process. Eighty-two males and 66 females with a wide age range (18–60 years) and a wide educational background completed the CMPT. Results revealed that none of the multitasking measures (accuracy, total time, total distance covered by the avatar, a prospective memory score, and a distractor management score) showed any sex differences. All effect sizes were *d* ≤ 0.18 and thus not even considered “small” by conventional standards. The findings are in line with other studies that found no or only small gender differences in everyday multitasking abilities. However, there is still too little data available to conclude if, and in which multitasking paradigms, gender differences arise.

## Introduction

When 488 participants from various countries including the US, UK, Germany, the Netherlands, and Turkey were asked “Do you think there are gender differences in multitasking?”, roughly 57% across all countries answered “yes” (Szameitat, Hamaida, Tulley, Saylik, & Otermans, 2015). When further prompted which of the sexes is better, ca. 80% of those that answered “yes” said “women” (Szameitat et al., 2015; for similar findings in a German sample alone see Strobach & Woszidlo, 2015). These studies prove the existence of a gender^1^ stereotype in multitasking favoring females, which appears to be more endorsed by women and its magnitude varies considerably across countries (Szameitat et al., 2015). On the other hand, it also suggests that about half of the people across all countries (i.e., 43%) do not think that there is a sex difference in multitasking.

By contrast, gender stereotypes with respect to verbal and spatial abilities are much more pronounced. For example, when participants were asked to rate the probability that a person is male or female and the only available information participants had about this person was that he/she “can imagine abstract objects and rotate them mentally in all directions”, 76% of respondents indicate that such a person is rather a man, 2% that it is rather a woman, and 22% indicate an equal probability that it is a man or a woman (Hirnstein, Andrews, & Hausmann, 2014). In turn, when given the information that this person “can easily remember names of guests on a party” or “speaks three different languages fluently”, 75 and 76% of participants, respectively, believed this person to be female—and only 5 and 2% believed this person to be male (Hirnstein et al., 2014). These findings are based on a German sample, but similar gender stereotypes have been reported in the UK (Hausmann, Schoofs, Rosenthal, & Jordan, 2014), Italy (Moè, Meneghetti, & Cadinu, 2009), and Turkey (Halpern and Tan, 2001). Such gender stereotypes regarding spatial and verbal abilities, however, reflect well-documented behavioral differences. For example, men reliably outperform women—on average—in mental rotation, the ability to imagine abstract objects from different perspectives. (e.g., Linn & Petersen, 1985; Voyer, Voyer, & Bryden, 1995; Zell, Krizan, & Teeter, 2015). Women outperform men—on average—when remembering a list of words or other verbal content (e.g., Catani et al., 2007; de Frias, Nilsson, & Herlitz, 2006; Herlitz, Nilsson, & Backman, 1997; Lowe, Mayfield, & Reynolds, 2003) or when generating as many words as possible under time pressure that fulfil a certain criterion, as in verbal fluency tests (Hyde & Linn, 1988; Hirnstein, Freund, & Hausmann, 2012; e.g., Hausmann et al., 2009; Scheuringer, Wittig, & Pletzer, 2017). Effect sizes of these cognitive sex differences vary between Cohen’s *d* = 0.30 for verbal fluency (Hyde & Linn, 1988) and *d* = 0.50–1.00 for mental rotation (Linn & Petersen, 1985; Voyer et al., 1995; Zell et al., 2015; Geiser, Lehmann, & Eid, 2008; Hirnstein, Bayer, & Hausmann, 2009; Moè, 2012; Moè and Pazzaglia, 2010). Many spatial and verbal abilities, however, do not show a male and female advantage, respectively (Hyde, 2014; for review Halpern, 2012; Miller & Halpern, 2014). Gender stereotypes with respect to verbal and spatial abilities are thus gross over-simplifications and -generalizations but at least partly grounded in reality, a phenomenon that has been claimed to be valid for stereotypes in general (Jussim et al., 2016). However, does this also apply to the gender stereotype about better multitasking abilities in females?

### Previous findings on behavioral sex differences in multitasking

Multitasking is an important everyday ability (for a recent overview see the editorial of a special issue by Poljac, Kiesel, Koch, & Müller, 2018) and a broad construct that can be conceptualized and assessed in various ways (e.g., Künzell et al., 2018). Burgess (2015) argues that there are at least two distinct types of multitasking, one in which two or more tasks are carried out simultaneously such as in dual task paradigms, termed “concurrent multitasking” (originally coined by Salvucci & Taatgen, 2008), and another type in which two or more tasks are carried out sequentially, termed “serial multitasking”. More specifically, in serial multitasking participants *alternate* between different tasks that vary in terms of priority, difficulty, and duration. Moreover, according to Burgess (2015), this alternation is interleaved. That is, the tasks cannot be accomplished in pure sequence but one needs to shift “back and forth” between them. Indeed, many everyday life activities are of a serial multitasking nature such as cooking, shopping, or working. So far, only a handful of studies have specifically investigated sex differences in multitasking abilities and the experimental paradigms that they used, as well as their findings, are inconsistent. In general, there seem to be three lines of findings. The first does not find any sex differences in multitasking abilities. For example, in a re-analysis of a study presented in 2010 (Watson & Strayer, 2010), where participants carried out an auditory–verbal task while in a driving simulator, males and females performed equally well when comparing dual task to single task performance (Strayer, Medeiros-Ward, & Watson, 2013). Similarly, no sex differences were observed in a study where participants carried out a driving task on a computer while simultaneously performing activities such as dialing a number on a mobile phone or reading out directions (Paridon & Kaufmann, 2010). Both studies employed concurrent multitasking paradigms.

At odds with the stereotype of a female superiority, a second line of findings yields a male advantage. Mäntylä (2013), for instance, employed a task in which participants had to perform three counting tasks simultaneously (e.g., press a button when the computer presents a multiple of 11) together with an *n*-back task. In two experiments, males had higher accuracy rates than females, but the advantage was largely mediated by spatial abilities (as measured by mental rotation performance). That is, if spatial abilities of male and female participants were matched, the sex difference in multitasking would disappear. Similarly, Hambrick, Oswald, Darowski, Rench, and Brou (2010) found a small male advantage (around *d* = 0.35) when performing a memory, arithmetic, audio monitoring, and visual monitoring task concurrently. This advantage was accounted for by the participants’ experience with playing video games. While both studies tested concurrent multitasking skills, Logie found a small to medium male advantage with *d* = 0.51 (R. Logie, personal communication, February 13, 2018) in the accuracy score of a paradigm that rather assesses serial multitasking, the Edinburgh Virtual Errands task (Logie, Trawley, & Law, 2011). Here, participants navigate through a three-dimensional computer environment and carry out prospective memory tasks. The focus of this study, however, was not sex differences in multitasking, but multitasking alone and, thus, it is likely that the male advantage did not arise from better multitasking abilities per se, but from generally better navigation skills (e.g., Persson et al., 2013). For this reason, the vast majority of studies that specifically investigate sex differences in multitasking use tasks that did not show sex differences before.

A third line of findings is in accordance with the stereotype that females excel in multitasking. For example, Ren et al. (2009) found that females showed less interference than males in a Flanker task when it was nested in a Go/No Go task, while no sex differences emerged when the Flanker task was carried out alone. However, one may question whether the Go/No Go task is in fact a task, or merely a signal for whether to execute the only actual task (the Flanker task). Thus, it is unclear to what degree this paradigm represents true multitasking. Nevertheless, in line with these findings, Stoet, O’Connor, Conner, and Laws (2013) found a female advantage in the most extensive study on sex differences in multitasking so far. In their first experiment, participants were asked to press either a left or right button depending on which stimulus was presented and in which condition it was presented (i.e., whether they had to attend to the shape or filling features of the stimuli). Participants generally responded more slowly when the two conditions switched rapidly as compared to performing only one condition. This performance reduction, however, was less marked in females. In a second experiment, participants were asked to complete three paper–pencil tasks comprising simple arithmetic, a key search task, and a map task within 8 min. Participants could freely choose how much time they would devote to each task. In addition, they received a phone call within this period where they were asked general knowledge questions. Females performed significantly better in the key search task (*d* = 0.49), while no differences emerged in the other tasks. Stoet et al. (2013) tentatively concluded that “woman are better than men in some types of multi-tasking (namely when the tasks involved do not need to be carried out simultaneously)” (p. 9). In other words, the authors suggested that a female advantage rather exists for serial but not necessarily for concurrent multitasking. However, they called for further studies to test their hypothesis.

Taken together, the findings regarding sex differences in multitasking abilities are rather inconsistent. The heterogeneity of the findings is not surprising given the heterogeneity of the methods that were used in those studies, for instance, the different tasks and different types of multitasking (i.e., serial or concurrent multitasking) that were assessed. However, apart from inconsistent methods and a general lack of empirical findings, there seem to be at least three further issues that make it difficult to discern whether there truly is a female multitasking advantage: first, many studies in this field used rather abstract multitasking paradigms (see also the comment by Stoet et al., 2013). This facilitates controlling for confounding variables but at the same time it is unlikely that the gender stereotype about females’ alleged superior multitasking abilities arose from how males and females handle the kind of tasks employed in scientific experiments. Most likely, the gender stereotype will have arisen from observing everyday situations and, thus, it is more likely to find these sex differences in everyday scenarios. Second, everyday scenarios typically involve serial multitasking (for review Burgess, 2015) and there is a clearly defined set of criteria for such scenarios (Burgess, 2000, 2015): they comprise (1) multiple, discrete tasks that (2) are interleaved but (3) carried out one at a time. (4) Unforeseen interruptions and problems occur and (5) there is no direct signal indicating when it is time to return to an already running task (delayed intentions). The tasks (6) differ in terms of characteristics, priority and length of time, (7) targets are self-determined, and (8) there is no immediate feedback. Many of the studies that are described above, however, do not meet one or more of these criteria. Third, previous studies on sex differences in multitasking often recruited student populations, making it difficult to generalize findings (see also the criticism by Stoet et al., 2013).

### The present study

We have previously devised a computerized task in a three-dimensional environment with the specific aim to test participants’ multitasking ability in everyday situations—the computerized meeting preparation task (CMPT, Laloyaux et al., 2014). The CMPT was originally conceived to assess the multitasking abilities of patients with schizophrenia, who were found to have profound multitasking difficulties (Laloyaux et al., 2014). However, given the issues with previous studies outlined above, we believe that the CMPT is very useful in the context of studying sex differences in multitasking abilities: First, unlike rather abstract paradigms, the CMPT has high ecological validity. At the same time, however, it allows reasonably well for controlling confounding variables due to its computerized nature. Second, the CMPT was specifically constructed based on the features of everyday multitasking situations as identified by Burgess (2000, 2015) and is thus solidly based in theory. Third, to validate the CMPT, participants were tested from different age groups and with different educational backgrounds, thus providing us with a more representative sample than, for example, a typical student population.

In summary, despite bold claims that “All the available research agrees: men’s brains are specialised. Compartmentalised. […] a man’s brain is configured for one thing at a time […]” while “A woman’s brain is configured for multi-tasking performance.” (Pease and Pease, 2001, pp. 69–70), the empirical evidence for a behavioral sex difference in multitasking is sparse and inconsistent, in particular, when it comes to serial multitasking abilities. The present study sought to add data to the discussion of whether the alleged female superiority exists by assessing males’ and females’ performance in an already established everyday situation multitasking paradigm (Laloyaux et al., 2014). Given the heterogeneous findings so far, we had no specific hypothesis as to whether a sex difference exists.

## Methods

### Participants

In total, 149 participants were included in the present study. None of the participants had a psychiatric or neurological diagnosis and none had first-degree relatives with schizophrenia or bipolar disorder. All were familiar with computers. One participant had to be excluded due to an unusually low accuracy level (23.8%) on the CMPT, which was six standard deviations below the mean (*M* = 91.0%) for all 149 participants. Of the remaining 148 participants, 82 were male and 66 were female. In addition to the CMPT, participants’ familiarity with video games and computers and their estimated IQ was assessed with a self-developed questionnaire and the French version of the National Adult Reading Test (FNART), respectively (further details are provided below). Finally, participants indicated their level of education (as measured by the number of successfully completed years of obligatory and higher education schooling). Demographic information for males and females with respect to age, level of education, NART IQ, familiarity with video games and computers is provided below in Table 1.

**Table 1:** Overview of sample with means of level of education, familiarity with video games and computer score, and NART IQ

	Age	Level of education*	Familiarity with video games and computers***	NART IQ
Females (*n* = 66)	32.6 (12.2)	13.3 (1.7)	12.9 (3.0)	107.5 (5.9)
Min–max	18–60	9–17	7–18	93.6–118.3
Males (*n* = 82)	33.2 (10.6)	12.7 (2.1)	15.1 (2.8)	106.1 (7.3)
Min–max	18–60	6–17	8–18	85.9–122.9

Values in brackets denote standard deviation. Asterisks indicate significant sex difference based on independent *t* tests ***p < 0.001; **p < 0.01; *p < 0.05

Independent *t* tests with sex as the dependent variable and age in years, level of education, the familiarity with video games and computers score, and the NART IQ as dependent variables revealed no sex differences in age, *t*(146) = 0.33, *p* = 0.739, and NART IQ, *t*(146) = 1.24, *p* = 0.217, but females had a slightly higher level of education, *t*(146) = 2.14, *p* = 0.034, while males indicated higher familiarity with video games and computers, *t*(146) = 4.59, *p* < 0.001.

Participants were recruited via word of mouth and received no incentive for participation in the study. The study was approved by the ethics committee of the Faculty of Psychology, Speech and Language Therapy, and Education—University of Liège (Belgium). All participants gave written informed consent to participate in the study and all methods conform to the Code of Ethics of the World Medical Association (Declaration of Helsinki).

### The computerized meeting preparation task

**Fig. 1:**
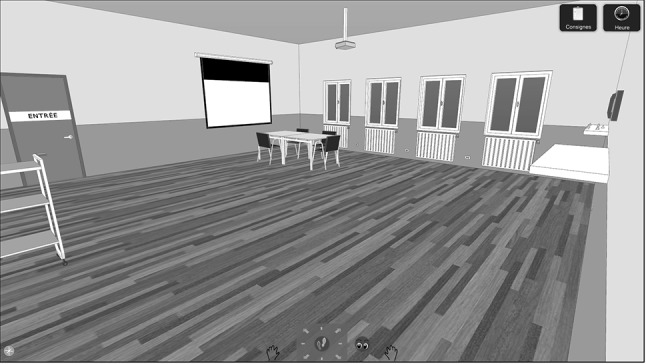
Screen shot from the computerized meeting planning task depicting the main room in which the meeting takes place

The CMPT was first described in Laloyaux et al., (2014); an updated version has been used in a recently accepted article (Laloyaux, Van der Linden, Nuechterlein, Thonon, & Larøi, in press). Table 2 illustrates how all eight criteria for everyday multitasking situations that have been put forward by Burgess (2000) were implemented in the CMPT. In brief, participants are asked to prepare a room for a meeting in an office setting in a three-dimensional computer environment (see Fig. 1). In a learning phase, participants are familiarized with how to operate their avatar and how to perform task-relevant actions such as grasping and transporting items. Participants begin with easy actions and, upon successful execution, advance to more complex actions. Participants receive feedback when errors occur and are asked to repeat the action until it is correctly performed. They proceed only when all actions have been performed successfully.

**Table 2:** Overview of characteristics of an everyday multitasking situation and its implementation in the CMPT

Characteristic of everyday multitasking	Implementation in the CMPT
Many tasks	The CMPT requires the completion of several main tasks (e.g., preparing the table for the meeting, follow prospective memory instructions, deal with interruptions) that rely upon many smaller tasks (consult the instructions, pick and place the correct items, move the cart, open doors, move around the room, look around the room, detect the missing chair, order a chair using the telephone, deal with the interrupting phone call, put the coffee on time, give the camera to an avatar, etc.)
Interleaved sequence	Completion of the tasks requires interleaving actions. For example, place items on the cart in the kitchen area, then consult the instructions, place other items on the cart, check if everything is correct, plan the completion of the next action, pick the cart, drop the cart near the table, go back to the instructions, go to the door because Chantal just arrived, go to get the camera and give it to Chantal, go back to the table to continue placing the objects, check the instructions, etc.
One task at a time	It is not possible to do many of the tasks at the same time. For example, it is not possible to read the instructions and place objects on the table at the same time; or it is not possible to pick items and simultaneously answer the telephone; further, it is not possible to plan the completion of the next action and to simultaneously check if the correct items are on the table due cognitive overload
Interruption and unexpected outcomes	There is a chair missing, the telephone will ring to signal a change in the desired drinks, someone will come to the door. In addition, participants can encounter more individual problems such as picking incorrect items or not remembering what to give to the avatar
Delayed intentions	The CMPT requires participants to put coffee on the table at a specific time, to give the camera to an avatar, and to remember to change the drink for one of the participants
Differing task characteristics	The CMPT involves many tasks of different nature, for example, there are some easy and short tasks such as putting the pencils on the table or more difficult ones such as selecting the correct nametags among the distractors or planning the completion of the next action. Some tasks have a high priority compared to others, (e.g., giving the camera to the avatar versus finding the missing chair). In addition, placing the items on the table involves different cognitive processes than moving around the environment, planning the completion of the next action, or recollect the intentions in memory
Self-determined targets	Participants define themselves what constitutes adequate performance as they determine when the task is completed
No feedback	Participants receive no feedback during the task

After the learning phase, participants are told that the meeting preparation phase will begin. First, a video is shown to familiarize participants with the environment: This consists of the main meeting room, in which a screen, a projector, tables, chairs and the name tags of the people attending the meeting can be found (including name tags of people who do not attend the meeting as distractors). In addition, there is a cart to transport items and a telephone that can be used to call different people (e.g., the secretary, the police) and to order items. Adjacent to the main meeting room is a kitchen and a storage room, which contain items that are needed for the tasks as well as distractor items. After the video, participants are told that it is 9:30 am and that a meeting will begin in 30 min, and that they have to prepare the meeting because the secretary got sick. Participants were given the following instructions: “The meeting begins at 10:00 am but do not waste time to prepare the room; carefully respect the instructions, only put the required objects on the table; the moderator has to be placed in front of the video projection screen and requires a laptop computer; the assistant has to be placed next to the moderator; every guest requires a pencil, a notepad, a name tag, and a chair—including the moderator; if the telephone rings or if someone asks you at the door, you have to answer; finally, when you feel that you have finished, please ensure that the cart is empty and that it is placed where you first found it, and then exit the room”. In addition, there is a list with the names of the guests at the meeting and their preferred drinks during the meeting. Throughout the entire meeting preparation phase, participants can access the instructions, the name list, and a clock. In addition, participants had to remember two prospective memory instructions that were given orally at the beginning of the task without the possibility of writing them down: “Give the camera to Chantal (an avatar) when she arrives.” and “Put the coffee on the table at 9:40 am.”. Finally, there were two distractors: a chair was missing that had to be ordered via the phone and the phone rings during the meeting preparation phase informing the participant that one of the guests wants another drink. The task was designed so that the level of difficulty can be adjusted by modifying the number of guests, prospective memory instructions, and distractors. For the present study, the standard level of difficulty proposed by Laloyaux et al. (in press) was used, which takes into account all the characteristics of multitasking activities without being too long or too difficult. With these settings, the vast majority of participants complete the task well before the designated 30 min. In case participants need longer, nothing particular happens. They just continue with the task until they exit the virtual room to indicate that they have finished the task.

The following variables were calculated for the meeting preparation phase of the CMPT: (1) accuracy (in %): the overall accuracy for the CMPT calculated as accuracy (%) = (Total_correct_ − Total_errors_)/42 × 100. Total_correct_ is the sum of all correctly performed actions and errands with a maximum of 42. That is, presence of five chairs (5 points), five note pads (5 points), five pencils (5 points), five name tags (5 points), one laptop (1 point), five drinking vessels (5 points), three types of drinks (3 points), as well as correct drink/name associations (5 points), correct location of the moderator and assistant (2 points), correct placement of the laptop (1 point), that the camera is given to Chantal (1 point), that the cart is empty at the end of the task and is stowed away in right location (2 points), a missing chair is ordered (1 point), and the coffee is placed at 9:40 am (1 point if the coffee is placed at 9:40 ± 1 min error margin). Total_errors_ is the sum of (1) all extra pertinent items placed on the table (e.g., six pencils instead of five = 1 error); (2) all non-pertinent items placed on the table (e.g., a stapler, a microphone, biscuits); and (3) all unnecessary phone calls (e.g., ordering incorrect items or calling inadequate recipients). For example, placing six chairs would award 5 points for the Total_correct_ score and 1 point for the Total_errors_ score. Further multitasking measures were (2) total time (in min): the time it took participants to complete the task (higher values indicate lower performance); (3) Total distance (in meters): the total distance participants covered with their avatar (higher values indicated less efficient and thus poorer performance).

In addition, we computed two further indicators that reflect specific aspects of multitasking performance: (4) prospective memory score: sum of all correctly performed actions that had to be remembered for some point in the future and that could not be looked up in the written instructions (i.e., putting coffee on the table at 9:40 am, giving Chantal the camera, and changing the drink after the phone call). The maximum score was three (higher values denote better performance). (5) Distractor management score: sum of all correctly performed actions that require dealing with interruptions or noticing deviance (i.e., ordering the missing chair, placing it, and changing the drink after phone call). The maximum score was three (higher values denote better performance).

### Familiarity with video games and computers questionnaire

Familiarity with video games and computers was assessed with an in-house questionnaire (Laloyaux et al., 2014, in press) composed of 6 questions asking participants to indicate the last time they used a computer or played with video games, the frequency of using a computer and playing with video games, their level of comfort with the utilization of a computer mouse, and finding their way in a virtual environment while playing with video games. Each question is scored on a 4-point Likert scale. The familiarity with video games and computers score is simply derived by adding the points together.

### National adult reading test (NART)

Participants’ IQ was estimated using the FNART (Mackinnon & Mulligan, 2005; Nelson & O’Connell, 1978). In this test, participants are required to read out 33 irregular words, that is, words that cannot be directly decoded using the grapheme–phoneme conversion. The estimated IQ is then calculated based on the number of incorrectly pronounced words.

### Procedure

Participants were assessed in a quiet environment. They first completed the demographics questionnaire and the questionnaire about familiarity with video games and computers. Subsequently, they performed the CPMT, followed by the FNART.

### Statistical analyses

The five dependent variables were not normally distributed based on Kolmogorov–Smirnov tests (all *D* ≥ 0.12, all *p* ≤ 0.001) and visual inspection yielded skewness towards high performance, particularly for accuracy, as well as for the prospective memory and distractor management scores. We nevertheless opted for parametric instead of non-parametric procedures because (1) samples as large as the one in the present study are better placed to deal with skewness than non-parametric tests (Frost, 2015) and (2) parametric procedures provide greater power to detect statistically significant sex differences. We carried out independent t-tests with sex as the independent variable and accuracy, total time, total distance, prospective memory score, and distractor management score as dependent variables. Nevertheless, we additionally computed Chi-square tests for prospective memory and distractor management scores since the narrow range (0–3) could also qualify for categorical data. Cohen’s *d* is provided additionally to ease comparison with other findings. Finally, we ran univariate ANCOVAs for each dependent variable with familiarity with video games and computers and level of education as covariates to control for possible confounders. Here, effect sizes are provided as partial eta squared (*η*^2^).

## Results

The independent *t* test did not yield significant sex differences for accuracy, *t*(146) = 0.40, *p* = 0.689, total time, *t*(146) = 1.04, *p* = 0.302, total distance, *t*(146) = 0.38, *p* = 0.703, prospective memory score, *t*(146) = 0.81, *p* = 0.419, and distractor management score, *t*(146) = 0.09, *p* = 0.927. Means, standard deviations and Cohen’s *d*s are provided in Table 3. When Chi-square tests were used, there was neither a significant association between sex and prospective memory score, *χ*^2^(2) = 2.21, *p* = 0.575, nor between sex and distractor management score, *χ*^2^(3) = 0.81, *p* = 0.847.

**Table 3:** Multitasking performance for five indicators in the CMPT with means (and standard deviations)

	Accuracy (%)	Total time (min)	Total distance (m)	Prospective memory score (max. 3)	Distractor management score (max. 3)
Females	91.7 (10.1)	15.3 (5.6)	194.0 (80.2)	2.45 (0.61)	2.36 (0.97)
Males	91.1 (10.2)	14.4 (4.5)	199.0 (77.6)	2.54 (0.61)	2.38 (0.94)
Cohen’s *d*	+ 0.07	− 0.18	+ 0.06	− 0.15	− 0.02

For accuracy, prospective memory, and distractor management higher values indicate better performance, while for total time and total distance lower values indicate better performance. Cohen’s *d* is always signed such that positive and negative values indicate better female and male performance, respectively

For the univariate ANCOVAs, we first confirmed that the homogeneity of familiarity with video games and computers and level of education did not significantly deviate between males and females for accuracy, total time, total distance, prospective memory, and distractor management scores, all *F*(1, 142) ≤ 2.07, all *p* ≥ 0.152. None of the five multitasking indicators yielded any significant sex differences when controlling for familiarity with video games and computers, and level of education, all *F*(1, 144) ≤ 1.11, all *p* ≥ 0.295; all *η*^2^ ≤ 0.01. Familiarity with video games and computers was found to have a significant effect on total time, *F*(1, 144) = 9.14, *p* = 0.003, *η*^2^ = 0.06, and total distance, *F*(1, 144) = 4.84, *p* = 0.029, *η*^2^ = 0.03. Simple Pearson product-moment correlations showed that higher familiarity with video games and computer scores were significantly associated with lower total time, *r* = − 0.24, *p* = 0.003, and a trend towards lower total distance, *r* = − 0.15, *p* = 0.065, thus indicating better performance. Familiarity with video games and computers did not have significant effect on accuracy, prospective memory, and distractor management scores, all *F* ≤ 0.66, all *p* ≥ 0.416; all *η*^2^ ≤ 0.005. Level of education did not have a significant effect on any of the five multitasking indicators, all *F* ≤ 2.22, all *p* ≥ 0.139; all *η*^2^ ≤ 0.015.

## Discussion

The presence of a seemingly widespread gender stereotype regarding females’ superior multitasking abilities (Szameitat et al., 2015) would suggest the presence of a behavioral equivalent. However, so far it is unclear if and under which circumstances a female advantage arises. The present study tested multitasking abilities in an everyday scenario computer paradigm and did not find any indication for a sex difference in a range of measures, regardless of whether familiarity with video games and computers and level of education had been controlled for. The obtained effect sizes were *d* ≤ 0.18, and thus, by conventional standards lower than “small” (*d* = 0.20). One might argue that the CMPT is too easy and ceiling effects could have masked potential sex differences. Indeed, the accuracy levels were fairly high with an overall accuracy mean of *M* = 91.4% (SD = 10.1) across all 148 participants. However, while it was relatively easy to accomplish most of the tasks since the instructions were always available, the speed (total time) and the efficiency (total distance) by which the tasks could have been completed allow for substantial variation. Moreover, there were no sex differences with respect to prospective memory and distractor management where instructions were not always available.

As Stoet et al. (2013) noted, the nature of the multitasking paradigm might be a crucial factor that determines whether a sex difference emerges. Specifically, they proposed that women are better “when the tasks involved do not need to be carried out simultaneously.” (Stoet et al., 2013, p. 9). Thus, women would be better at serial but not concurrent multitasking. However, the present findings do not support the hypothesis of a universally better female performance in serial multitasking. The CMPT is evidently a serial multitasking paradigm and does not yield a sex difference. On the other hand, one needs to bear in mind that our null findings are insufficient to refute the hypothesis of Stoet et al.: Possibly, there is a female (or male) advantage in the CMPT, but we may not have had sufficient power to detect it. With an effect size of *d* = 0.18, the largest we found, one would need at least 766 participants in total (50% females) to reliably find a sex difference (based on an independent *t* test, *α* = 0.05, power = 0.80, one-tailed, as calculated with G*Power, Faul, Erdfelder, Buchner, & Lang, 2009). In turn, the current sample had enough statistical power to detect a sex difference with *d* = 0.41 or larger (based on a sensitivity analysis with *α* = 0.05, power = 0.80, one-tailed; G*Power, Faul et al., 2009). Thus, if a sex difference exists in the CMPT, it is small.

Alternatively, women are not universally better at serial multitasking but their advantage is limited to a specific sub-component of serial multitasking paradigms, that is, switching between tasks. In Experiment 1 in Stoet et al. (2013), participants needed to switch rapidly between the set of rules that they needed to apply, however, within the same task framework. Similarly, in Ren et al. (2009) females showed lower switching costs when a Go/No Go paradigm was nested in a Flanker task. Based on the present study and the available empirical findings to date, at least we cannot rule that there is a female advantage in serial multitasking paradigms that require rapid shifts between tasks.

Our findings are in line with two other studies that did not find sex differences in concurrent multitasking in everyday settings (Strayer et al., 2013; Paridon & Kaufmann, 2010). Both studies tested participants in a driving simulator or with a driving task while carrying out an auditory–verbal task (Strayer et al., 2013) or activities such as dialing a number on a phone or reading out directions (Paridon & Kaufmann 2010). As far as everyday multitasking scenarios are concerned, only Stoet et al. (2013) found a female advantage in Experiment 2. Specifically, females outperformed males in the “Key Search Task” while no sex differences emerged in a Map search task and simple arithmetic questions in Experiment 2. There were also no sex differences in the general knowledge questions that were asked when participants picked up the phone call that was meant to distract them. As pointed out above, this was a serial multitasking paradigm where participants could freely choose if and when to tackle each task. Although it is still too early to draw firm conclusions, there appears to be a trend according to which sex differences are small to non-existent in paradigms that assess both serial and concurrent multitasking abilities in everyday situations.

Some studies have revealed a male advantage in concurrent multitasking that chiefly arose from better spatial abilities (Mäntylä, 2013) or more experience with computers in males (Hambrick et al., 2010). We, too, found that males had more experience with computers and that this was correlated, albeit weakly, with multitasking performance. More specifically, the total time and the total distance in the CMPT was lower in participants with greater levels of familiarity with video games and computers, while no association was found for accuracy-related measures. This could reflect that participants with more computer experience might find navigating the avatar easier, which allows for greater speed and movement precision (and hence shorter total time and total distance). However, this does not necessarily help with completing the required tasks correctly and, hence, familiarity with video games and computers has little impact on accuracy scores. In any case, the positive effect of computer experience did not lead to a significant sex difference in the CMPT.

Due to the lack of empirical findings on behavioral sex differences in multitasking, it is not surprising that even less is known about their potential neuronal mechanisms. It is fairly established that serial multitasking draws especially on the rostral prefrontal cortex as evidenced by lesion studies (Dreher, Koechlin, Tierney, & Grafman, 2008; Roca et al. 2011; Burgess, Veitch, de Lacy Costello, & Shallice, 2000) or functional near-infrared spectroscopy (Langhanns & Müller, 2018). Concurrent multitasking has been linked to areas within the prefrontal cortex, but also parietal, temporal, occipital and cingulate areas in a training study with healthy individuals (Takeuchi et al. 2014). However, data on sex differences in multitasking in general is sparse. In a recent functional magnetic resonance imaging (fMRI) study, 20 males and 20 females performed two types of dual tasks (i.e., concurrent multitasking). In one dual task condition, they matched upper- and lower-case letters in addition to a one-back task. In another dual task condition, they had to mentally rotate arrows in addition to a one-back task (e.g., Tschernegg et al., 2017). The authors found that females had stronger activations in the inferior frontal gyrus during the verbal dual task, an area typically associated with speech production (Hickok & Poeppel, 2007). Men had stronger activations in the precuneus and nearby visual areas during the spatial dual task. Interestingly, no behavioral differences were observed (Tschernegg et al., 2017). This demonstrates that even when there are no sex differences behaviorally, the underlying neuronal activity can differ between males and females, suggesting that males and females process multitasking differently. Perhaps, this reflects different cognitive strategies when handling multitasking situations. However, this is pure speculation at this point.

### Limitations

A few limitations need to be considered when evaluating this study. The first is obviously that such null findings rarely help to confirm or refute hypotheses or open up new lines of ideas. Second, the CMPT provides a wealth of data and possible variables that were not reported here: for example, time spent in a certain area, the number of times when the instructions or clock was checked, or the time that elapsed before the instructions were checked for the first time, etc. We focused on the most relevant measures for the sake of clarity, but also did not find significant sex differences for other measures. At any rate, the CMPT provides multiple possibilities for future investigations. For those who are interested, it is freely available in Dutch and French at http://www.meetingpreparationtask.com. As pointed out above, accuracy rates may be subject to ceiling effects. The current CMPT version already allows for changing the number of guests, prospective memory instructions, and distractors. Future studies should thus increase the difficulty level.

Third, we based the present study on Burgess’ definition of everyday multitasking situations (2000) and his distinction between serial and concurrent multitasking (2015). It should be noted, however, that the distinction is not discrete in the sense that multitasking paradigms must either fall into the serial or concurrent category. In other words, there may be a certain degree of overlap between the two. In particular, Burgess (2015) suggested that concurrent multitasking may be embedded in serial multitasking and that being able to complete two tasks at the same time may facilitate completing a serial multitasking activity (e.g., placing the objects on the table while planning the next task). However, this issue has never been examined and future studies are thus required to examine the relationship between serial and concurrent multitasking. Moreover, given that the CMPT is specifically designed to test everyday multitasking, which is mostly of a serial nature, we still deem it appropriate to classify it as a paradigm that chiefly (but not exclusively) assesses serial everyday multitasking.

Fourth, unlike classic dual task designs the CMPT does not contain a single task or baseline condition that can be contrasted with the dual task/multitasking condition. Thus, in theory, it is possible that women are, in fact, better at multitasking than men, but that this difference does not emerge because women perform more poorly on single task elements and, as a result, the overall sex difference in the CMPT is zero. However, we are not aware of male advantages in basic memory and executive functions that are required for the CMPT. A male advantage in computer games has been controlled for statistically, and a male advantage in navigation does not seem relevant, as the environment (three rooms) is too small. If any, the well-documented better verbal memory in females (Catani et al., 2007; de Frias et al., 2006; Herlitz et al., 1997; Lowe et al., 2003) might help them remembering the instructions better. In addition, some of the cognitive processes that underlie serial multitasking abilities might only become relevant when two or more tasks need to be coordinated (Burgess et al., 2000), making it difficult to create an adequate single task condition baseline for the CMPT or serial multitasking abilities in general.

Finally, the specific cognitive strategies adopted by participants during the CMPT were not examined. For example, how they handled the interrupting phone call and how it affected the main tasks. Possibly, these strategies differ between individuals (and between men and women) and impact the way the task is carried out, but not necessarily the final result. Future studies are required to further examine this issue.

### Conclusion

The contribution of the present study is that we add empirical data to a small but steadily growing body of evidence and that we corroborate previous results that behavioral sex differences in everyday multitasking seem negligible. There are suggestions according to which females outperform males in paradigms where participants have to quickly switch between tasks (Stoet et al., 2013). However, we feel that the only justified—though not very satisfactory—conclusion at this stage is that it is unclear whether females are indeed better at multitasking abilities in general or any specific form of multitasking. The only way to solve this issue is by providing further empirical data.

## Footnotes

1 We use the term “gender” when referring to stereotypes and “sex” when referring to cognitive differences between males and females. This rationale is based on Diane Halpern’s considerations in her book “Sex Differences in Cognitive Abilities”, where she argues that cognitive differences between males and females arise from a complex interplay of biological, psychological, *and* socio-cultural factors. These factors would be so intertwined that they are practically inseparable. Hence, it would not be logical to distinguish between biology (sex) and social environment (gender). Since “gender” is often used in contexts where it exclusively refers to social environment, while “sex” at least covers biological aspects and since most studies in this field meant to categorize female and male participants based on biology, “sex” seems more appropriate in this context. In line with Halpern, however, we would like to emphasize that this does not necessarily reflect a superiority of biological factors.
